# A Case of Angular Anchylosis of the Knee Joint, Treated on Dr. J. R. Barton’s Plan by Platt Burr, M.D.

**Published:** 1844-06

**Authors:** Andrew R. Kilpatrick

**Affiliations:** Woodville, Mississippi


Art. II.—*A Case of angular Anchylosis of the Knee Joint, treated on Dr. J. R. Barton's plan by* Platt Burr, M. D. Reported by Andrew R. Kilpatrick, M. D., of Woodville, Mississippi.

Among the new operations in surgery, that of Dr. Barton, for *Anchylosis*, occupies a conspicuous place, and if that gentleman had done nothing more, this alone would have been sufficient to enrol his name with the worthies who preceded him, and hand it down to the latest posterity. Cases which were formerly abandoned as irremediable, and the subjects of which were suffered to drag out a miserable and helpless existence, or subjected to the remorseless quackery and torture of "*Bonesetters*," can now be relieved and placed in a situation comparatively easy and independent.

The following case occurred in the practice of my friend, Dr. Platt Burr, of Cheneyville, La., who has requested me to report it for him:

Sam, a negro man, æt. 40 years, of stout frame, and good general health, while engaged, on the 12th December, 1840, in hewing a log, accidentally let the broad axe slip, and the corner of it struck the left knee with considerable force, on the inner side, and penetrated the side of the head of the tibia close to the patella. The wound was not more than an inch long, and soon healed up, although there was slight pain in the part. By exposure and active exercise, the knee was swollen rapidly to a very large size, and that, together with the pain, disabled him for work and forced him to take his bed. Medical aid was called in and efforts were made to check and remove the pain and inflammation, but without success. The whole limb became enormously enlarged, suppuration came on, and a great quantity of pus was discharged at three several openings, above and below the knee. The purulent discharge ceased, the limb was reduced in size, the muscles were flaccid, and the skin was loose, dry and crusty, so much so that when rubbed or thumped it emitted a sound like that of coarse brown paper or dry raw hide. The knee still continued unnaturally large, and the skin on it was tight and glossy. As he suffered less pain when the leg was flexed, he was permitted to keep it in that position so long that it became fixed; the synovial fluid and membranes were destroyed, the femur, tibia and patella all united firmly together as if they were a continuation of one bone, and from the firm adhesion which had taken place considerable force would have been required to disunite them. When he was seated no one could perceive that any deformity existed; but on his assuming the erect position, his leg and foot projected backwards at a right angle with the other leg. His master despaired of his ever being cured, or benefitted by any surgical assistance, and had concluded to apprentice him to a shoemaker that he might be of service to him in that way. Before acting on this determination, however, he requested Dr. Burr to examine him, when, from the appearance of the limb, the general health of the patient, and his robust constitution, he concluded he could perform Dr. Barton's operation with safety, and Mr. Brown, the master, consented to a trial of it, the patient himself being willing to submit to any thing which promised an amelioration of his condition.

After the patient was duly prepared to undergo the operation, Dr. Burr sent for me to be present and assist him; Dr. Ingersoll and several gentlemen of the vicinity were also present. The day was quite cold and raw. As before stated, the thigh was flaccid, skin loose, knee large, the patella projecting boldly out and adhering firmly to the condyles and tibia.

On the afternoon of the 8th December, 1841, being one year, wanting four days, from the time of the injury, the patient was placed on a table with his legs hanging down over the end, his body and arms being held by assistants. I attended to the parts to be operated on, adjusting the skin, &c. The first incision was made by commencing at a point opposite to the upper and anterior margin of the external condyle of the femur, and passing obliquely upwards across the front of the thigh, terminated on the inner side. The second incision commenced also on the outer side, about three inches above the first, and passing obliquely downwards across the front of the thigh, terminated at the point of the other incision, thereby forming an acute angle. The integuments, the extensor muscles, part of the vastus externus and internus and the crureus were divided by these incisions. The triangular flap was then dissected up from the femur close to the condyles and the soft parts on either side were pressed down out of the way, so as to admit of the free use of the saw. The only knife employed in all the operation was the common scalpel.

The flap then being turned up and held out of the way, a common amputating saw was used, and by inclining it, a wedge-shaped piece of the femur was taken out, about two and a quarter inches at the base, and three lines at the apex. The femur was not completely divided by the saw, as was suggested by Dr. Barton, but about three lines were left undivided, which was fractured by forcibly drawing the leg back, and the spiculæ thereby formed answered the purpose admirably of keeping the extremities of the bone in their proper place; and moreover the danger of injuring the popliteal artery was thereby avoided.

The time required for the operation was about five minutes. There were three small arteries divided which required ligatures; when they were secured the flap was returned to its place, secured by a few sutures, the ends of the ligatures were brought out and fixed, and adhesive strips and light compress and bandages completed the dressing.

Before moving the patient from the table, the limb was placed at the same angle of flexion it was at before the operation, on a double-inclined plane similar to Amesbury's apparatus, with a moveable cross piece to regulate and fix its angularity, and a foot piece with a shoe attached to it. The plane was rendered comfortable by long bags of carded cotton, suited to the shape of the parts and so adjusted as to prevent pressure or friction upon the popliteal region. The limb was kept in that angular position for some weeks, until it was thought the spiculæ and asperities of the bone were either absorbed or covered by deposite. The sutures and ligatures of the arteries were properly attended to and removed. Adhesion of the soft parts progressed rapidly and regularly to complete re-union, with little pain or trouble.

By means of the brace on the apparatus the limb was gradually straightened until brought very nearly straight, but agreeably to the suggestion of Dr. Barton, a very slight angularity was maintained intentionally, in order to prevent his heel, in walking, from striking against any obstacle or inequality which might be in the way. The double-inclined plane was then thrown aside and the limb placed in the ordinary straight box used in common fractures, where it was kept for better than three months, at the expiration of which time he was allowed to move about on a crutch, as the bones had knitted together pretty well.

In the month of June, 1842, he could walk about on his leg very well, even without the use of a staff, and in fact went to work. But on the 15th of July of the same year, in attempting to mount a ladder he missed his footing, and in the fall broke his leg again at the point of operation. As he had complained of his foot hurting in the instep, at the metatarsus and toes, especially when he walked much, and when standing on logs chopping, when it was reset the last time it was placed in the straight box without any angularity, being quite straight. No bad results followed this fracture, and in less than two months he was able to be up and about again, and could walk very well without crutch or staff. It has been thought by some who were acquainted with this case, that the adhesion of the bones was thereby rendered more firm. During the whole time his general health was unexceptionable.

On the 15th April, 1844, I saw Sam at his master, Mr.

W. R. Brown's, on Bayou Bœuf, Louisiana, busily engaged spading up the garden. He told me that he could hoe and chop with as much ease as he ever did; that ploughing was not irksome to him; and Mr. B. said he was nearly as serviceable as ever. There is no pain or swelling after a day's labor, but his limb appears as sound as ever.

May, 1844.

